# A Comparison of Real and Virtual Laboratories for Pharmacy Teaching

**DOI:** 10.3390/pharmacy10050133

**Published:** 2022-10-14

**Authors:** Jennifer Schneider, Chelsea Felkai, Irene Munro

**Affiliations:** 1 School of Medicine and Public Health, College of Health, Medicine and Wellbeing, University of Newcastle, Callaghan, NSW 2308, Australia; 2 School of Biomedical Sciences and Pharmacy, College of Health, Medicine and Wellbeing, University of Newcastle, Callaghan, NSW 2308, Australia

**Keywords:** virtual laboratory, real laboratory, blended learning

## Abstract

New approaches to teaching and learning in the tertiary setting offer students flexibility for learning and, in a pandemic, suggests ways to provide learning when face-to-face delivery cannot be conducted. Courses that contain a hands-on laboratory component can be resource intensive in terms of equipment, staff, and facilities, thus more difficult to deliver when hands-on laboratory work is precluded. This study developed two virtual laboratories that could be completed online and, using a crossover design, evaluated student learning outcomes from virtual and real laboratory activities for 57 students. It also gained student feedback on their learning experiences. Overall, student knowledge increased significantly for each topic after completing either the virtual or real laboratory activities. However, no significant difference in learning was observed when outcomes from virtual or real laboratories were compared. Feedback from students indicated that most students found online modules easier to follow, they provided better background information, and would be revisited, but real laboratories were more interesting. Reinforcing learning, understanding, and remembering processes were reportedly similar for both, indicating no negative impact when a virtual laboratory was used. This study provides supporting evidence for the use of virtual laboratories where the focus is on learning concepts and not on student proficiency at operating laboratory equipment.

## 1. Introduction

Advances in digital technology have provided opportunities for educators to create blended learning where online interactive material that students can access both on and off-campus is combined with face-to-face classroom activities. In a 2021 survey of students in Australia and New Zealand, blended learning was the most preferred overall method of learning, and 25% of the students surveyed also said they would switch institutions for a better technology experience [1]. The Flipped classroom model, in which traditional lectures are replaced with online material enabling students to access the work at a time and place that best suits them and is combined with interactive face-to-face sessions to review and apply knowledge, has been adopted by educators across many disciplines [2]. Using online material with or without some face-to-face activities has also become an essential part of tertiary education during the COVID-19 pandemic. However, when a course contains a significant component of hands-on laboratory activities, essential for reinforcing concepts and learning, the reduced ability to conduct face to face laboratory sessions presents a challenge. Even before the pandemic, universities faced difficulties in providing resources for laboratory-based learning. As the number of students attending universities grows, so does the demand for specialised facilities for laboratory teaching. Building and equipping new laboratories, updating old ones, and maintaining and replacing laboratory equipment are costly [3,4]. When in use, laboratories require a lot of space, and only small numbers of students can occupy a room at any one time so running the laboratories is labour intensive [5,6], and set up and tear down time can be longer than the actual experiment performance time [7,8,9]. Approaches using technology may help to address these challenges. However, replacing hands-on laboratory activities with interactive online material raises the question of whether this approach is as effective and acceptable to students as hands-on laboratory activities. It is important that laboratory-based education maintains its effectiveness while reducing costs [10].

In the early stages of the Pharmacy degree training, students often receive training in pharmaceutics and dosage form design as part of their curriculum. Experiments demonstrating basic concepts in topics such as Rheology and Surface Tension require specialised equipment. This equipment is expensive [4]. It requires the purchase of several of the same instruments to enable all groups in the same laboratory session to perform experiments over only a few weeks each year. Equipment must then be maintained and stored until the following year. The experiments are designed to teach concepts, but students do not need to become proficient in using this equipment for future professional duties. When the demand for laboratory space and facilities became an issue in the Bachelor of Pharmacy program at our university, there was an opportunity to develop and evaluate the use of a virtual online laboratory activity for these topics. The COVID-19 pandemic has also reinforced the need for online learning when population lockdown resulted in many universities suspending face-to-face learning activities, and moving to online learning [11].

To compare the educational outcomes achieved with real, hands-on and virtual, online laboratory sessions, our research team conducted a study to compare these approaches. This study was approved by the Human Research Ethics Committee at the University of Newcastle, Australia, approval number, H2013-0151.

## 2. Materials and Methods

Two topics where the experimental activities involved the use of specialised equipment were selected for the study. One topic was based on experiments in Rheology and involved the use of a Brookfield^®^ viscometer (Brookfield Engineering Laboratories Inc., Middleboro, MA, USA) to examine the rheological properties of pharmaceutical fluids. The other topic was based on surface and interfacial tension and involved the use of surfactants and a tensiometer. A virtual online laboratory (VL) module and a hands-on real laboratory (RL) module were developed for each topic. The experiments performed in each of these topics were identical in the VL modules and the RL modules.

### 2.1. Development of VL Modules

The VL modules were created using the software Softchalk^®^ (SoftChalk LLC, San Antonio, TX, USA), and Udutu^®^ UDUTU, Victoria, BC, Canada). The modules consisted of pages introducing the theory underpinning the experiments to be performed followed by a description of each experiment. A video was produced for each experiment, taken at an angle from which students would be operating and viewing output as if they were using the equipment hands-on. The video camera was set up to ensure that the manipulations and the output such as the data screen on the device were captured. The recorded video was edited using iMovie^®^ software (Apple, Los Altos, CA, USA) and titles were added for each experiment. Students were able to slow, stop, rewind and zoom the videos. Instructions were provided to students on collecting the data as the experiment progressed. Instructions on the plotting of data and calculations at the end of experiments were also provided. Short quizzes for students to self-check their learning were built into the modules. These were not scored but provided the students with the correct answers to support their learning. The developed modules were exported as SCORM files and embedded into the learning management system (Blackboard). Students were able to access these modules in the laboratory and at home and were able to work alone when using this module.

### 2.2. Development of RL Modules

The RL modules for the Rheology and Surface Tension practicals involved developing written instructions in a laboratory workbook provided to each student. These workbooks contained pages introducing the underpinning theory (similar to the online module) and instructions on solutions to be prepared and steps to be performed in each experiment. Students referred to the printed workbook during the experiments and worked in groups of 3 or 4. Two viscometers and two tensiometers were available in the laboratory. For Rheology experiments, the liquids to be tested were pre-prepared by a laboratory technician. Students were shown how to operate the equipment by a laboratory supervisor and written instructions were also supplied for each instrument. Students operated the equipment and recorded data manually in their workbooks. Students used the recorded data to generate plots, perform calculations and explain the outcomes of observation experiments. A supervisor was available to assist with any technical difficulties that arose and to reduce the risk of equipment damage. Over one year, the viscometers and tensiometers would be used for about 2 to 3 weeks and then placed in storage for use in the following year. Each year, the equipment would be checked and serviced prior to the laboratory sessions.

### 2.3. Experimental Design

**Figure 1:**
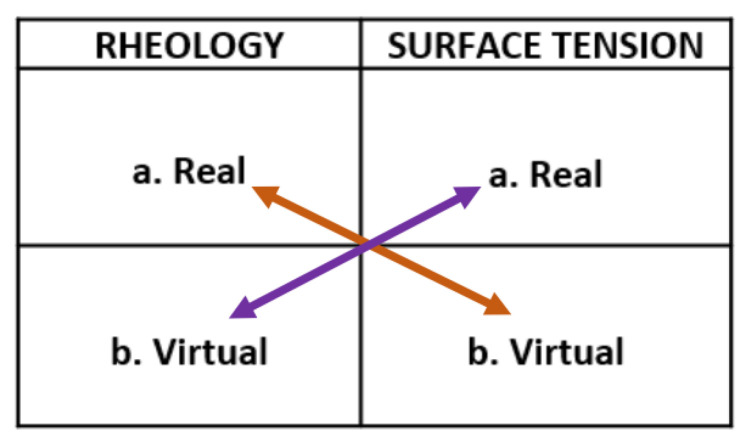
Procedure for laboratory work.

All students in the first year Introduction to Pharmacy course participated in a combination of a VL accessed in the laboratory and a RL. The sequence of laboratory sessions occurred over a two-week period, with students completing one session each week. They were randomly assigned to participate in a real Rheology session and a virtual Surface Tension session (shown by the orange arrows) or a real Surface Tension session and a virtual Rheology session (shown by the purple arrows) in Figure 1.

The virtual module could be accessed by the students on-line in the laboratory. The real modules were conducted face-to-face in the laboratory and experiments included paper-based information and instructions with the practical for the students.

### 2.4. Evaluation of RLs and VLs

Before commencing the laboratory work, students completed an in-class quiz to determine baseline knowledge of Rheology and Surface Tension. For Rheology, there were three questions, one of which was in four parts, and for Surface Tension, there were six questions, one of which was in two parts. Each question or part of a question was assigned 1 mark. As students were not expected to know the answers in the baseline quiz, for each question the response ‘I don’t know the answer’ was included to discourage students from guessing. After completing their laboratory experiments, the students repeated the same quiz again with the response ‘I don’t know the answer’ removed. To enable comparison of correct quiz responses between baseline and post laboratory learning, students identified their quiz responses with their own self-generated code so that the quiz results could be matched but were anonymous. Students were advised that the results from these quizzes would not be included in their course assessment.

After completing both a VL and a RL, students were also asked to indicate their preferred laboratories for different learning experiences from the choices ‘Virtual’, ‘Real’ or ‘Same’ using a 5-question survey. For each question, there was an open-ended response asking students to indicate the reason(s) for their choice.

### 2.5. Data Analysis

Student results from the pre- and post-test quizzes were collated and analysed using SPSS (IBM Corp. Released 2017. IBM SPSS Statistics for Windows, Version 25.0 Armonk, NY, USA). Thematic analysis was used to identify common themes/responses to individual preferences for laboratories that support learning.

## 3. Results

A total of 62 students completed the modules. Five students who were absent on the day of their RL were able to make up the work for that laboratory by completing a second VL experience, but their data could not be included in the study. Thus, the final number of students who completed both a VL and RL in this study was 57 students.

### 3.1. Pre- and Post-Test Results

All students completed the same pre- and post-tests. The results were then grouped into Rheology and Surface Tension questions by VLs and RLs. Paired sample t-tests were conducted to determine whether there was a significant difference between the pre- and post-test results within each of these four modules. To determine whether there was a significant difference in student scores for VLs or RLs, independent sample t-tests and correlations were conducted. Significant improvements were recorded for all four modules (*p* < 0.001) (Table 1). Twenty-seven students completed the Rheology VLs, recording an improved mean score of 3.87, SD 2.41. Thirty students completed the Rheology RLs, recording an improved mean score of 2.6, SD 2.6. For Surface Tension, 30 students completed the VLs, and showed an improved mean score of 3.85, SD 3.11, and 27 students completed the RLs, recording an improved mean score of 3.83, SD 2.23. Pearson’s correlations were calculated between the pre- and post-test scores for each module. All test scores were moderate in the range of 0.44 to 0.6, indicating that there were differences between students in the degree to which they had improved (Table 1).

### 3.2. Comparison of VL and RL Results

The final test results for the VL and RL for Rheology and for Surface Tension were calculated and compared to determine whether the differences were significant. For Rheology, the mean difference between final results for the VLs (*n* = 27) and the RLs (*n* = 30) was 1.27 and not significant (*p* = 0.062). For Surface Tension, the mean difference between the final results for the VLs (*n* = 30) and the RLs (*n* = 27) was 0.017 and not significant (*p* = 0.981) (Table 2).

### 3.3. Preferred Laboratories for Learning

**Figure 2:**
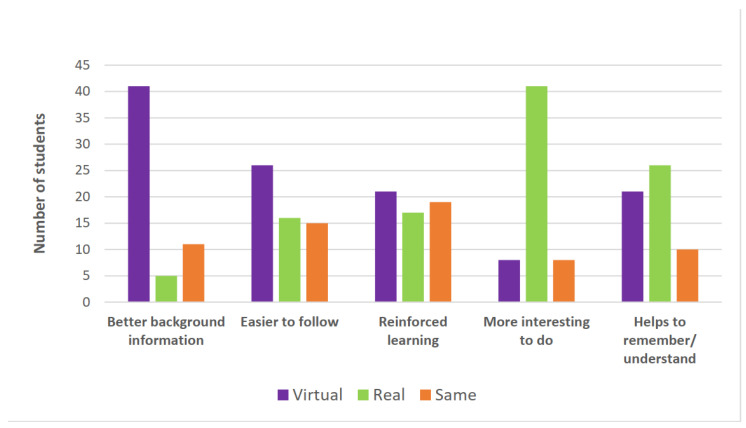
Student preferences for laboratories for learning.

Data from the 5-question survey for student preferences for laboratories for learning was collated into three options, VLs, RLs, or the Same, with students varying their choice of laboratory depending on the perceived learning support provided. The distribution of student responses is shown in Figure 2; the number and percentage of students is shown in Table 3. All 57 students responded to each of the five questions.

### 3.4. Reasons for Student Preferences for Choices of Laboratories

Following each question asking students to indicate their preferred laboratory for learning, there was an open-ended response option for students to indicate the reason(s) for their choice. 27 students provided open-ended responses for all questions, 6 provided no responses, while 24 provide some responses. Recurring keywords and phrases from the responses were grouped into themes by laboratory type. The identified themes are described below.

- VLs comprised two themes, “Readings and materials which were detailed, easy to understand and supported with quizzes”, and “Readings, given in advance, and personal control to pause and replay and work at own pace”.
- RLs comprised one theme, “Hands on involvement, easier to remember”.
- Same comprised one theme, “No difference”.

**Figure 3:**
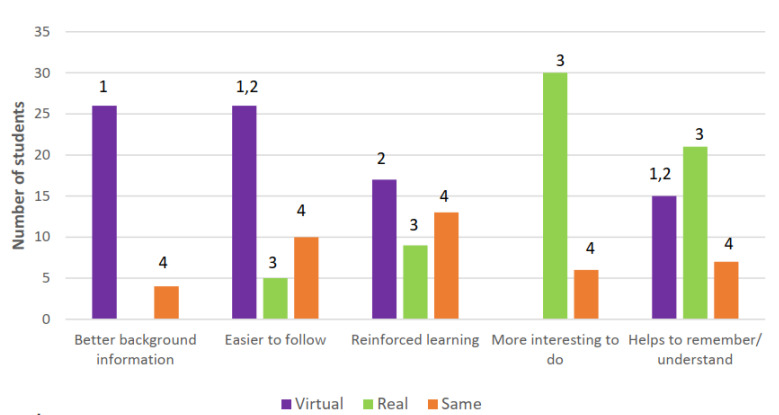
Reasons for student preferences for laboratory choice by themes.

The number of open-ended responses grouped by the classifications of VL, RL or same and identified themes for each of the 5 questions are shown in Figure 3.

Themes:

1. Readings and materials: detailed, easy to understand, quizzes;
2. Readings and personal control: pause/repeat, own pace;
3. Hands-on involvement;
4. No difference.

For ‘better background information’ and ‘easier to follow’, the majority of students selected VLs (Figure 3). Feedback from the open-ended questions for VLs stated that the readings, which were provided prior to viewing the laboratory experiments, were very informative and easy to understand and explained the reasons behind the processes and concepts, and that they had to be read to answer the presented questions. Additionally, the readings enabled them to control the pace of their learning, by pausing and repeating the video when needed.

Identifying the laboratories for ‘reinforced learning’ and ‘remembering/understanding’, a similar number of students selected VLs and RLs (Figure 3). VLs were preferred for the repetition of information in the readings which students claimed helped to reinforce their learning and being able to work at their own pace which gave them time to absorb and remember the information. Students who preferred RLs for reinforcing their learning claimed that they did so because the hands-on physical experience made it easier to understand what was happening.

The majority of students selected RLs as the most interesting laboratories to complete (Figure 3) saying that hands on experience was more interactive, more fun and maintained interest. According to one student, ‘you can see the science’, and another, ‘you get to physically hold it’.

For each of the five learning experiences, a number of students reported that, for them, there was no difference between VLs and RLs (Figure 3). A number of individual comments did not link to one of the themes and these are not included here. Not all students provided a response. Space was also made available for students to make additional comments about their use of the VLs and RLs. Sixteen students made additional comments, but these did not differ from the comments already made and included above. A final question asked students whether they would access the VL again to review the material if it was continually available. Twenty-nine students indicated that they would, four students indicated that they would not, and seven students said ‘maybe’. The remaining 17 students did not respond.

When students had completed the laboratories, pre- and post-laboratory quiz, and the preference survey, they were offered the opportunity to experience the alternative VL or RL so that they were not disadvantaged in any way. Five students took the opportunity to do so.

## 4. Discussion

To address a growing need for flexible learning options for students, this study was designed to develop VLs for student learning and to compare student outcomes and acceptability with RLs. Thus, the same topics were delivered in two different ways. The questions are, do VLs and RLs support different ways of learning, and do they produce similar or different learning outcomes?

A comparison of the difference from pre- to post- test results in both RLs and VLs for Rheology and Surface Tension showed a significant difference in mean scores suggesting that student knowledge improved significantly with both types of laboratories. When the final test results for VLs and RLs were compared within the topics of Rheology and Surface Tension, the differences were not significant. This would suggest that there is little difference in learning through RLs or VLs. Both create an active learning experience enabling the students to be cognitively active in constructing their own knowledge. It has been suggested that in this constructivist approach to learning it is the cognitive activity of the learner that matters and not whether the laboratory apparatus is virtual or real [6,12].

These findings are consistent with the work of others where studies have been conducted in different countries at different levels of education and in different disciplines. For example, no significant differences in student achievements between VLs and RLs were observed in physics laboratories with 90 university students in Palestine, [13], or with 80 pre-service school teachers in physics laboratories in Turkey [12], or in chemistry laboratories with 45 high school students in Germany [14].

Chan [15] reports that there is little evidence to show that VLs have worse outcomes compared to RLs, and says that there are many advantages associated with using VLs [15]. The benefits identified by the students in the current study are consistent with the findings reported in other studies. These include providing for individual learning needs such as students working at their own pace, being able to revisit the recordings to reinforce learning and understanding, and time saved with virtual experiments [16]. As there are no safety concerns with VLs [17], another benefit is a reduced need for supervision enabling students to develop self-sufficiency skills [18].

However, the majority of the students in the current study indicated that the RLs were more interesting to do, with many claiming that hands-on work helped them to better understand and remember the processes of the experiments. Additionally, RLs are important as VLs do not provide students with the opportunities to develop the practical technical skills required in the workplace [11,16] While developing technical skills through RLs would certainly be important in other professional training settings, in the case of pharmacists, use of equipment such as viscometers and tensiometers would not be part of normal day to day activities and proficiency in their use would not be required. Glassey [19] suggests that although VLs cannot replace the physical interaction of students with equipment, the students can still become familiar with the procedures and equipment operation through observation [19] It has also been shown that students have been able to accurately manipulate laboratory apparatus after watching their laboratory partner conducting the procedure and students that have learned a skill in the virtual environment are able to successfully perform the skill in real life [20]. A plausible explanation for equivalent effectiveness for VLs and RLs is when images of real apparatus are used in VLs, physical materials are not necessary [12].

Addressing the question of specific ways of learning for the different laboratories, students might prefer RLs and be disinclined to complete VLs in the belief that they do not match their preferred learning style which they believe is conducive to better outcomes. However, the concept of individual learning styles has been questioned [21] According to Claxton and Ralston [22], a learning style is the consistent way a student responds to and uses stimuli for learning [22]. While many students identify with a particular ‘style’ of learning and may believe that this is linked to better outcomes, it would appear that most students use multiple learning strategies. When using the VARK questionnaire to identify the way students learn, Hussman [23] reported that despite identifying a personal style, over two-thirds of the 400 students involved in the study used multiple learning styles, and that no style or combination of styles resulted in better outcomes [23]. Additionally, for the one-third of students who did use their identified personal style for learning, there was no apparent academic benefit [23] Studies have not shown that students learn better using a self-reported learning style [21].

Rather than particular learning styles it has been suggested that cognitive load is a primary constraint to learning effectively. According to Clark et al. [24] reducing total cognitive load during learning increases the availability of working memory and improves the learning process [24]. Aspects of the virtual modules are mostly designed in a way that can reduce cognitive load, with students enabled to work at their own pace, to pause and repeat the presentation of information as well as repeating the experiments when there is a need [15,25]. Consideration can also be given to introducing other strategies at the design stage, for example simultaneously presenting some information in visual mode and other information in auditory mode enabling learners to use both processing channels thereby reducing the load compared to using only one channel [13]. Although, there were no differences observed in learning outcomes between VLs and RLs in the short term in this study, it is possible that in the long-term the effects of reduced cognitive load could improve learning outcomes through retention of information.

## 5. Limitations

A limitation of this study is that while we demonstrated student learning was improved soon after the laboratory activities, no measure of long-term retention of learning was performed. Additionally, while VLs are lower cost for teaching large numbers of students, which would be most beneficial for less developed countries, student reach could be compromised by the availability of computers or hand-held devices such as tablets or phones.

## 6. Conclusions

This study demonstrated that it was feasible for academic staff to develop VLs using readily available software and hardware. Being able to provide VLs offers a potential solution when there may be time or resource restrictions, or in situations such as a pandemic, where RL work is curtailed. The results observed here showed that VLs did not negatively impact student learning, producing similar outcomes, which is an important consideration when changing from traditional RLs to VLs. While students do see benefits in RL activities, VLs do offer the ability for students to revisit and redo the laboratory activities and to watch the experiments differently by slowing and rewinding the videos.

## Figures and Tables

**Table 1:** Differences between pre- and post-test results for Rheology and Surface Tension, on-line virtual and hands-on real laboratories.

						*p*	95 % CI		Correlation	
Group	*n*	Mean	SD	SEM	t	2-Tailed	Lower	Upper	r	*p*
Rheology:										
Virtual: Pre-test	27	1.667	2.38	0.46						
Post-test	27	5.537	2.13	0.41						
Difference	27	3.870	2.41	0.46	8.35	0.000	4.82	2.92	0.44	0.023
Real: Pre-test	30	2.467	2.86	0.52						
Post-test	30	5.067	2.08	0.38						
Difference	30	2.600	2.60	0.48	5.47	0.000	3.57	1.63	0.48	0.007
Surface Tension:										
Virtual: Pre-test	30	3.783	3.72	0.68						
Post-test	30	7.633	3.18	0.58						
Difference	30	3.850	3.11	0.57	6.79	0.000	5.01	2.69	0.60	0.000
Real: Pre-test	27	2.611	1.95	0.38						
Post-test	27	6.444	2.59	0.50						
Difference	27	3.833	2.23	0.43	8.94	0.000	4.72	2.95	0.55	0.003

**Table 2:** Differences between the final test results for Rheology and Surface Tension, on-line virtual and hands-on real laboratories.

						*p*		95 % CI	
Group	*n*	Mean	SD	SEM	t	2-Tailed	df	Lower	Upper
Rheology:									
Virtual	27	3.870	2.41	0.463					
Real	30	2.600	2.60	0.475					
Difference		1.270			1.905	0.062	55	0.037	2.577
Surface Tension:									
Virtual	30	3.850	3.11	0.567					
Real	27	3.833	2.23	0.43					
Difference		0.017			0.023	0.981	55	1.401	1.435

**Table 3:** Individual student preferences for laboratories for learning (*n* = 57).

Questions	Virtual	Real	Same	*n*=
	%/*n*	%/*n*	%/*n*	
1.Which lab provided better background information for your work?	72/41	9/5	19/11	57
2.Which lab was the easier one to follow?	46/26	28/16	26/15	57
3.Which lab reinforced your learning?	37/21	30/17	33/19	57
4.Which lab was the more interesting one to complete?	14/8	72/41	14/8	57
5.Which lab enabled you to understand and remember the processes?	37/21	46/26	17/10	57
	**Yes**	**No**	**Maybe**	*n*=
6.If access to the virtual lab was continually available, would you access it again to review the material	74/42	9/5	17/10	57

## Data Availability

The data presented in this study are available on request from the corresponding author. The data are not publicly available due to privacy and ethical reasons.
